# The bright side of social media information overload for anti-COVID-19 behaviors: a stimulus-organism-response framework

**DOI:** 10.3389/fpubh.2025.1556542

**Published:** 2025-04-14

**Authors:** Shang Meng, Lingling Zhong, Junwei Cao

**Affiliations:** ^1^School of Flight, Anyang Institute of Technology, Anyang, Henan, China; ^2^School of Business, Yangzhou University, Yangzhou, China

**Keywords:** information overload, norm activation theory, COVID-19 pandemic, prosocial behavior, epidemic prevention

## Abstract

Social media has an important impact on social management by communicating information that influences individual behavior; however, too much social media information can cause user information overload. The coronavirus disease 2019 (COVID-19) pandemic context provides an opportunity to analyze the impact of social media information overload on individuals’ prosocial behaviors in emergency events. In this study, the impact of social media information overload on people’s anti-COVID-19 behaviors, that is, behaviors that limit the transmission of COVID-19, was investigated by linking norm activation theory and information overload theory. Data were collected from 242 Chinese participants, and structural equation modeling was used for data analysis. The results showed that social media information overload positively influenced the awareness of consequences and the ascription of responsibility in anti-COVID-19 behaviors and that ascription of responsibility and awareness of consequences could activate people’s personal norms and eventually motivate people to engage in prosocial behaviors for epidemic prevention. These findings could extend the information overload concept to a public health emergency context and yield useful insights for world pandemic control.

## 1 Introduction

Social media is social, communicative, interactive, and reliable, which facilitates the communication of information, reduces losses and promotes social collaboration during disasters (1). Government communication on social media significantly influences public fear levels and serves as a tool to monitor emotional responses during crises (The role played by government communication on the level of public fear in social media: An investigation into the Covid-19 crisis in Italy). Social media can accelerate information disclosure, inform people about how to protect themselves and improve social collaboration (2). Although the investigation of social media information cannot replace all professional sector prevention efforts, public health professionals can use it to make quick and effective decisions (3). Therefore, we believe social media has an important role in controlling the COVID-19 pandemic.

The COVID-19 pandemic was the first international public health emergency since the emergence of the massively popular social media, which provides an unparalleled opportunity to analyze the impact of information overload due to social media in social crisis management. Social media has been helpful in managing this pandemic by communicating the situation to people in a timely manner, but the large amount of information on social media has also introduced many problems. The most prominent one is information overload. The overwhelming influx of crisis-related information during the COVID-19 pandemic has contributed to information overload, leading to cognitive fatigue, stress, and even news avoidance as individuals struggle to manage excessive and often contradictory information (News Avoidance during the Covid-19 Crisis: Understanding Information Overload) In the current COVID-19 pandemic, people chose social media as their preferred information source (4) and were inevitably affected by information overload. However, the effects of information overload during the COVID-19 pandemic remain complex (How Differential Dimensions of Social Media Overload Influences Young People’s Fatigue and Negative Coping during Prolonged COVID-19 Pandemic? Insights from a Technostress Perspective) Therefore, it is necessary to examine how information overload affects people’s anti-COVID-19 behavior (e.g., wearing a mask, actively cooperating with home isolation, not gathering, etc.).

As shown in Table 1, most current studies analyzed the impacts of information overload due to social media in the COVID-19 epidemic. For example, Valika et al. (5) pointed out that the massive dissemination of news, while enhancing the exchange of information among people in a pandemic, also contributed to the spread of lies and misconceptions, leading to information confusion and overload among the general public and creating unnecessary social panic, which may have a negative impact on controlling the spread of COVID-19. During the COVID-19 pandemic, information overload can lead to cognitive fatigue and avoidance behaviors among the public, ultimately reducing their willingness to engage in proactive actions against COVID-19 (Swiss residents’ information behavior perceptions during the COVID-19 pandemic: A longitudinal qualitative study) Accordingly, the majority of studies have concluded that information overload has negative consequences for people’s decisions to adopt anti-COVID-19 behaviors.

**Table 1:** Research on information overload in the context of COVID-19.

Source	Sample	Theory base	Methods	Dependent variable	Key findings
Cao et al. (54)	286 Chinese respondents	Push-pull-mooring framework	Survey	Dissatisfaction, Anxiety, Perceived policy, Subjective norms, Perceived switching costs, Switch intention	Information overload results in dissatisfaction, anxiety, increased perceptions of policy benefits, lower perceived switching costs, and ultimately leads to vendors switching to street vending.
Farooq et al. (81)	225 Finnish respondents	Protection-motivation theory	Survey	Self-efficacy, Response costs, and Willingness to isolate.	Cyberchondria and information overload affect individuals’ willingness to self-isolate through the threat and their coping
Hong and Kim (24)	627 Korean respondents	-	Survey	Heuristic processing, Systematic processing, and anti-COVID-19 behavioral intentions	Information overload positively affects individuals’ heuristic processing but negatively influences their systematic processing. Overall, information overload is found to be a factor for anti-COVID-19 behavioral intentions if the information is processed systematically.
Laato et al. (84)	211 Finnish respondents	Stimulus-organism-response framework	Survey	Cyberchondria; Self-isolate intention, and Intention to make unusual purchases.	Cyberchondria, caused by information overload, affects individuals’ intention to isolate themselves and to make unusual purchases.
Song et al. (60)	721 Chinese respondents	Stimulus-organism-response framework	Survey	Anxiety, Cognitive dissonance, and Information avoidance intention.	Information overload leads to anxiety and cognitive dissonance, thereby increasing information avoidance intention, which indicates that information overload is a negatively influences the adoption of COVID-19 preventive behaviors.
Valika et al. (5)	7,719 articles from the PubMed database	-	Conceptual	-	Information overload is a source for misconceptions, information confusion, and unnecessary social panic.
Xiao et al. (25)	150 Chinese respondents	Cognitive-affective personality system theory	Survey	Information anxiety and Impulsive buying	Information overload positively impacts information anxiety, which in turn leads to impulse buying.

The question arises whether information overload could enhance anti-COVID-19 behaviors during the COVID-19 pandemic? When negative information causes overload, individuals can misinterpret the information and make bad decisions, but if the information is positive, information overload can cause a positive change in an individual’s attitude (6). Although some evidence indicates that people become more selective and efficient in their information search when experiencing information overload (7), these positive effects of information overload have not been clearly stated. Based on the stimulus-organism-response (SOR) framework, a recent study examined several unusual consumer behaviors, it was revealed that information overload enhanced people’s cyberchondria (i.e., a person’s anxiety about their health that is created or exacerbated by using the internet to search for medical information) and this has led to negative responses to the epidemic (Exploring Antecedents of Cyberchondria During Pandemics: An Integration of Stress and Coping and SOR). Overall, to further inform scholars and policymakers, there is an urgent need to address the impact of information overload on anti-COVID-19 behaviors.

To summarize, a stream of related studies found that information overload have negative impact on the Covid-19 epidemic [Swiss residents’ information behavior perceptions during the COVID-19 pandemic: A longitudinal qualitative study; (5)]. However, in fact, research is still lacking on the positive impact of information overload in the epidemic, and information overload can lead to people not processing information according to their actual abilities or motivations but rather according to their own instincts, leading to uncertainty about whether positive epidemic prevention behaviors can be triggered. Therefore, this study aims to answer the following question: How does information overload affect citizens’ emotions and anti-COVID-19 behaviors?

**Figure 1:**
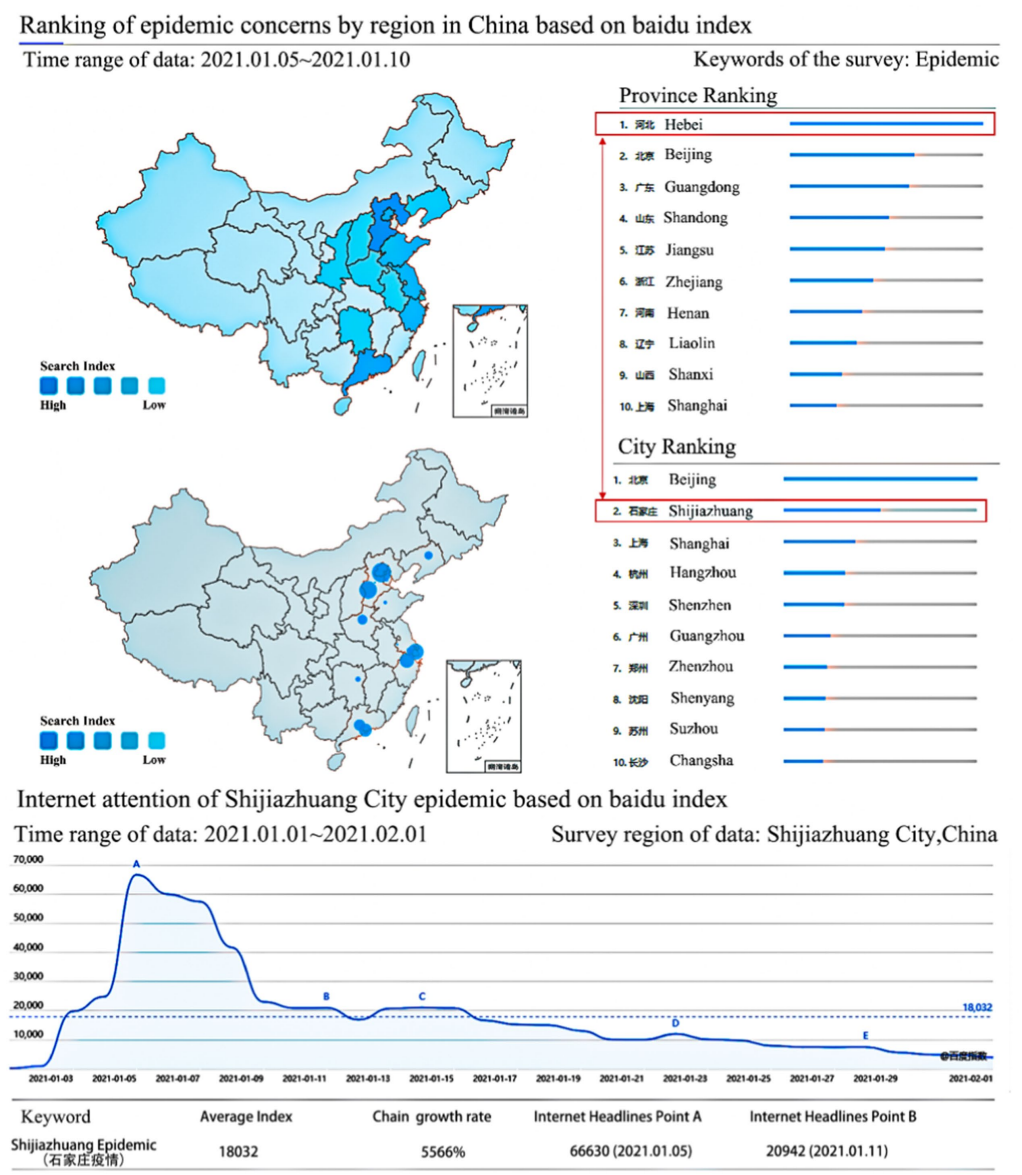
Search index of COVID-19 outbreak in Shijiazhuang city.

In January 2021, there was a sudden outbreak of the epidemic in Shijiazhuang, China. The Chinese government, in conjunction with companies, communicated various epidemic prevention information to the public instantly and effectively via the internet. Base on Baidu Index,^1^ after the COVID-19 outbreak in Shijiazhuang city, epidemic-related information on the internet increased by 5,566%, attention from mobile SNS apps increased by 6,861%, and most of these concerns come from Shijiazhuang city (please see Figure 1). During this period, the epidemic prevention department communicated much information to the community through the internet to improve the effectiveness of epidemic prevention; social media increased its influence by communicating the epidemic news; and local residents were eager to get relevant information to protect themselves by looking for information through the internet. So it is reasonable to believe that there may be information overload for Shijiazhuang residents during this time period. Information overload on social media may influence citizens’ emotions and anti-epidemic behavior in Shijiazhuang city. A very good opportunity has been presented to us to analyze the positive role of information overload in epidemic prevention.

To address this issue, a survey of Chinese respondents was conducted. By building a hypothetical model based on norm activation theory, the responses were analyzed. The contributions of this study are (1) to identify the possible positive role of information overload in social crises and extend the theory of information overload and (2) from the perspective of people’s emotions, introduce information overload into norm activation theory in order to explain how information overload affects prosocial behavior. These findings can provide new ideas for social crisis management, especially for anti-COVID-19 behaviors.

This paper is structured as follows. Section I provides the background of the study. Section II includes the theoretical background and hypotheses. Section III presents the research model and questionnaire for this study, and Section IV presents the data analysis. Section V summarizes and discusses the study’s findings, explains its contributions and limitations, and offers directions for future research.

## 2 Theoretical background and hypotheses

### 2.1 Social media in emergency management

Social media has a significant role to play in helping agencies and people manage social crises. Currently, people use social media for emergency responses in social crises (45%) much more than they download and use professional emergency apps (16%), and the level of social media used by people in social crises is increasing in many countries (8). An increasing number of scholars advocate the inclusion of social media in the management of information dissemination in social crises. Social media has significant advantages over traditional media in the management of social crises through media, as it is easier for relevant organizations to communicate with the public through social media and to infer user behavior through social media data (Social Media and Health Communication: A Review of Advantages, Challenges, and Best Practices). Social media can improve situational awareness by providing timely, actionable information about evolving disasters, and people’s reports of disaster developments through social media can alert society to specific issues that they need to be aware of in a crisis (9), understand and organize the spread of rumors (10), generate faster awareness of public crises (11), and influence convergent behaviors that are consistent in resisting disasters (12).

In social crises, the transmission of information through social media can be beneficial, but too much information from social media has been shown to have a negative effect on crisis management, especially information overload. When social media transmits too much information, it can be overwhelming and confusing, and one does not know what information is necessary (13), and limited by information processing capacity, most of the information may be ignored, influencing the individual’s decision-making. Information delivered by social media is also often brief and informal, and this information has no context; these single logical statements make it difficult for the population to understand, especially in large-scale emergencies, where more information is disseminated than usual, so that people are unable to find pertinent information effectively, let alone organize, understand, and act on this information, ultimately leading to action failures (9, 14, 15). Therefore, social media operators need to consider how to effectively control relevant information in a crisis so that the benefit of individual decisions is maximized (9, 13).

The information conveyed by social media has various such roles in social crisis management, but some uncertainties are involved. Some studies have confirmed that the use of social media in emergency situations varies depending on the national crisis management culture (16). Crisis management cultures have generally been divided into three types: state-oriented, individualistic, and fatalistic. In the state-oriented crisis culture, people believe that the extent of the crisis is determined by the environment and people, and that people can mitigate the crisis through relevant actions. They have a high level of trust in the state crisis management sector through social media information and instructions conveyed by the state crisis management department through social media, and highly comply with these instructions (16, 17). In individualist-oriented crisis cultures, people also believe that it is possible to reduce negative consequences and prevent crises by acting accordingly, but trust in government crisis management is not as high as in state-oriented ones, and their social media pages tend to show a higher perception of crisis response themselves, believing that they each have a responsibility to participate in the response (16, 17). Fatalistic crisis cultures consider the occurrence of crises unpredictable and unavoidable, an irresistible force of nature. People in these cultures have low trust in the media during crises and lack confidence in solving problems, and both expect the state authorities to take action but do not take seriously the information issued by the state in response to crises (16, 17). Therefore, it is important to fully understand the type of disaster culture of the affected population, which can help to develop strategies for information management in disasters.

Overall, the information presented through social media in social crises is not necessarily representative of the responses of the entire population, but they do represent a range of behaviors, ideas, and perspectives that can work alongside traditional crisis responses (15). For example, social media messages can make people aware of specific issues that increase perception of the state of affairs, create crisis awareness and ultimately specific crisis management behaviors (9, 11, 12), or may have a negative effect on crisis management due to the ambiguity of the information (14, 15). It can be considered a stimulation-organism-response mechanism in general, that is to say, different messages from social media give positive or negative stimuli to people in a crisis, which produce positive or negative emotions, and different emotions eventually trigger different behaviors of people to manage the crisis. However, at present, whether the emotions and behaviors triggered by different stimuli are positive or negative still need to be clarified. Especially in different social crises and different crisis cultures, this needs to be studied in a targeted manner. Therefore, this study will further explore, based on the stimulus–response mechanism, the specific effects of information overload on people’s prevention behaviors due to social media in this epidemic from the perspective of information overload.

### 2.2 Stimulus-organism-response framework (S-O-R)

The stimulus-organism-response (S-O-R) framework was first proposed by Mehrabian and Russell (18). The S-O-R theory was first applied to research in environmental psychology, but as research progressed, it was also widely used for studying information systems (19, 20). The S-O-R framework points out that some features of an individual’s environment can trigger cognitions and emotions that lead the individual to behave in a particular way (21). The S-O-R model consists of three aspects: the environment or stimulus that can elicit individual behavior and response (S), the responding organism (O), and the actual response (R). Stimulus (S) is the influence that can induce changes in individuals (22). Organism (O) refers to the emotionally and cognitively mediated state that emerges when an individual interacts with a stimulus, which reflects the process of regulation between the stimulus and the user’s response, where emotion reflects the emotion that the individual displays in response to the stimulus, and cognition is a mental state that refers to the process by which the individual acquires and processes the stimulus received in the brain. Response (R) is the individual’s approach or avoidance response to a stimulus (20). Approach responses are manifested as positive and optimistic behaviors in response to stimuli from a given environment, and avoidance responses are negative behaviors in response to stimuli from a given environment. Overall, S-O-R theory can integrate individual responses to explain an individual’s perceptions and emotions in response to exterior stimuli and consequent positive or negative behavior (23).

The S-O-R mechanism is well suited to explain the influence of social media information on people’s precautionary behavior in this outbreak. As social media platforms serve as spaces for individuals to engage with, interpret, and respond to health-related information during a crisis, the information shared on social media reflects diverse behaviors, ideas, and perspectives (The Role of Social Media Influencers in Public Health Communication: Case COVID-19 Pandemic)Information on social crises contains a wide variety of useful content, such as information about the loss of individuals and facilities, the collection of disaster relief resources, issuance of alerts, psychological support, etc. (13), and this information may then create stimuli for people who are unaware of the actual external situation due to their long-term confinement to their home. Negative characteristics, such as the stimulus of information overload (24), trigger panic and information confusion (5), and these emotions eventually lead to behaviors that are detrimental to epidemic preparedness, such as impulse buying, lack of epidemic preparedness, and information avoidance (24, 25). In this regard, social media is a good way to promote social awareness. Regarding positive characteristics, social media can stimulate people to deepen their perception of the situation by providing timely information about the disaster, making them aware of the issues that need attention (9), creating public crisis awareness more quickly (11) and eventually converging behaviors to fight the disaster (12). However, because crisis management cultures are different in homogeneous countries (16), negative stimuli from social media messages do not necessarily result in negative consequences, and positive stimuli do not necessarily result in positive consequences, which implies the need for specific analyses for specific disasters and countries.

### 2.3 Norm activation theory

Norm activation theory is designed to examine altruistic intentions and behaviors in a prosocial context (26). It is comprised of four main constructs that predict prosocial behavior: awareness of consequences (AC), ascription of responsibility (AR), personal norms (PN), and prosocial behavior. In norm activation theory, prosocial behavior refers to behaviors that benefit or promote harmonious relationships with others and is a general term that covers a range of positive social behaviors, such as giving help, cooperating, analyzing, and comforting (27). According to the theory, a person’s prosocial behavior or intentions are influenced by personal norms, and both consequence awareness and ascribed responsibility are factors that influence these norms. To determine the impact of individual responsibility on behavior, norm activation theory has been applied to environmental, psychological, and environmentally behavior research. People‘s Norm are activated when they become aware of the consequences of their unsocial behavior and feel responsible for it (26). This activates one’s moral obligations to perform or avoid certain behaviors (28). To prevent a harmful outcome, these norms determine whether a specific action should be taken by an individual (29).

The relationship between the factors in norm activation theory has been studied extensively, but the relationship between awareness of consequences, attribution of responsibility, and personal norms remains controversial. Some researchers argue that norm activation theory is a mediating model in which consequence awareness and attribution of responsibility indirectly influence prosocial behavior by influencing personal norms (30). Other scholars assert that the norm activation model is a moderating model in which consequence awareness and attribution of responsibility play a moderating role in personal norms influencing prosocial behavior (31, 32). However, it is possible to study prosocial behavior using both mediation and moderation models. For example, Zhang et al. (33) studied employees’ energy saving behavior using a mediated norm activation model. He and Zhan (34) investigated electric vehicle use in China by activating ethical norms using a mediation model. However, in general, mediator-type norm activation models can better explain prosocial behavior (35).

The present study concluded that awareness of consequences, ascription of responsibility, and personal norms in the norm activation model are consistent with the definition of organism (O) in the S-O-R framework. Mental states related to awareness of consequences, attribution of responsibility, and personal norms can be characterized as emotions expressed after individuals have acquired and processed stimuli from their environment. The phenomena of awareness of consequences and ascription of responsibility have been confirmed in many studies on the emotional state of an individual after receiving environmental stimuli (36–38). A prosocial behavior can be considered a response (R) in the S-O-R framework when the organism develops awareness of consequences, ascription of responsibility and personal norms in response to environmental stimuli.

The awareness of consequences is the awareness of negative consequences to other people or objects as a result of an individual’s failure to accomplish a corresponding goal (26). Individuals can use it to gauge their awareness of the negative consequences of their non-prosocial behavior, and it is often able to activate personal norms and initiate prosocial behavior (26). According to numerous studies, awareness of consequences can directly affect personal norms. For example, Han and Hwang (39) examined visitors’ decision-making through norm activation theory, and they found that personal norms were positively impacted by an awareness of consequences. An evaluation of how China’s haze pollution affected the purchase of energy-efficient appliances by Song et al. (40) revealed a positive effect of consequence awareness on personal norms.

Many studies also point to the positive impact of consequence awareness on the ascription of responsibility. He and Zhan (34) studied the behavior of people using electric vehicles in China based on norm activation theory and found a positive effect of consequence awareness on the ascription of responsibility. Wang et al. (41) studied information disclosure and residents’ behavior in waste sorting based on norm activation theory and a positive correlation was found between awareness of consequences, attribution of responsibility, and waste sorting.

Anti-COVID-19 behaviors are behaviors that benefit others or promote harmonious relationships with them and can be considered prosocial behaviors, such as helping in epidemic prevention, cooperating with society to take epidemic prevention measures, and analyzing the epidemic situation to adopt epidemic prevention behaviors that benefit themselves and society. Prosocial behaviors are influenced by the attribution of responsibility, awareness of consequences, and personal norms (30), so it is appropriate to use norm activation theory in this study.

During the current COVID-19 epidemic, the public learned about prevention practices through social media messages, which may have made them aware of the negative effects on society caused by the worsening of the epidemic (e.g., massive spread of COVID-19, the continued stagnation of social production, etc.), and such awareness of the consequences may have triggered a sense of personal responsibility to help prevent continuation of the epidemic, which ultimately activated personal norms. Based on the above description, this study proposes the following hypotheses:

*H1*: Awareness of consequences has a positive impact on personal norms.

*H2*: Awareness of consequences has a positive impact on the ascription of responsibility.

Ascription of responsibility is defined “the perception that one is responsible for the negative consequences of non-prosocial behavior” (35) and is a sense of responsibility that an individual has for the negative consequences associated with not performing the target behavior. A prosocial personal norm develops when people feel responsible for the consequences of their negative actions. Research has confirmed that the ascription of responsibility has a positive impact on personal norms. After analyzing passengers’ decision-making processes in terms of using environmentally friendly products during cruise trips, Han et al. (42) found that attribution of responsibility is positively correlated with the formation of personal norms. Rezaei et al. (43) combined norm activation theory and the theory of planned behavior to study farmers’ willingness to engage in integrated pest management and found that when farmers perceive a personal responsibility to carry out pest management, this sense of responsibility leads to a strong intention to engage in pest management. People may feel a strong ascription of responsibility and activate personal norms when they feel that they may have an impact on society because of their negative behaviors (e.g., not wearing a mask, participating in community activities, etc.). Based on the above description, this study proposes the following hypothesis:

*H3*: Ascription of responsibility has a positive impact on personal norms.

Personal norms refer to “the moral obligation to perform or avoid particular acts” (28). Personal norms are the closest variable to prosocial behavior in norm activation theory and can also be referred to as moral norms. When people are aware of negative consequences for others, they tend to assign responsibility for these consequences to themselves. Assigning responsibility thus activates personal norms, which determine whether they should act in a certain way to mitigate the negative consequences (44). Many studies have analyzed the relationship between personal norms and prosocial behavior: Van Der Werff and Steg (45) analyzed people’s concerns about energy based on norm activation theory and found that people are motivated to pay attention to energy issues by their personal norms. Zhang et al. (46) analyzed Chinese citizens’ intention to complain about environmental issues based on the mediation and moderation models in norm activation theory and pointed out that personal norms have a positive effect on people’s willingness to complain. Wittenberg et al. (47) analyzed the behavior of households using photovoltaic energy based on norm activation theory and pointed out that personal norms can influence people’s behavioral intentions toward photovoltaic energy use. Once people establish personal norms in the COVID-19 pandemic context, they may develop a sense of moral obligation to perform epidemic prevention behaviors, which leads to prosocial behaviors. Therefore, this study expects that activating personal norms has a positive effect on influencing individuals to carry out behaviors to prevent COVID-19. So, it is hypothesized that:

*H4*: Personal norms have a positive effect on anti-COVID-19 behaviors.

### 2.4 Information overload

‘Information overload’ was first used by Goodall and Gross (48). A classical definition of information overload is when the information processing demand exceeds the capacity of the information processing system (49). In most cases, information overload happens during the retrieval and interpretation of data, as well as during the decision-making process (49). Overload of information is caused not only by an overwhelming amount of information but also by stress, confusion, pressure, and anxiety that individuals can experience as a result of being exposed to it (50, 51). A person’s knowledge and experience can also affect the degree of information overload (52). Individuals have limited knowledge reserves and cognitive abilities. In cases of information overload, the individual’s cognitive ability exceeds the amount of information available, especially when the information is ambiguous and complex (53).

Social media is a major source of information overload. As information technology develops rapidly, people have access to information through social media, which generates hundreds of millions of pieces of information every day. However, a vast amount of information is not always necessary for people because it may generate information overload. Study have confirmed that SNSs give users too much information, making them feel bored and tired and causing various effects (54). Information overload from social media is not only widespread but also introduces new types of overload. There are three types of information overload from social media: information overload, communication overload, and system feature overload. Information overload occurs when people are exposed to more social media information than they have the capacity to process (49). Communication overload occurs when the demand for communication through social media exceeds the user’s ability to communicate (55).

The effect of information overload on an individual is social fatigue, confusion, difficulty prioritizing, impaired ability to use previous information, poor decision making, dysfunction, and anxiety (56). As a result of information overload, information seekers become anxious, depressed, and angry when the speed of information access exceeds their capacity to process it (57).

This study argues that information overload can be defined as a stimulus (S) in the S-O-R framework because information overload is considered a stressor that provides constant stress to the organism (O) (58, 59). In a study based on the S-O-R framework, information overload was defined as the stimulus of the environment, and the user’s sense of regret and fatigue were defined as the organismic performance, pointing out that users’ organismic response is significantly influenced by this information overload (19). Song et al. (60) used information overload as the stimulus (S) factor in the SOR framework to investigate consumer information avoidance behavior in the COVID-19 pandemic context. Information overload is perceived as a stressor that constantly stimulates users’ emotions by constantly presenting them with repetitive information about the epidemic, which continuously creates an awareness of its consequences and ascribed responsibility. People prefer to compare their behavior and performance with their friends on social media (61). The same may be true in an epidemic, as information overload may deepen the image of the severity of the situation, and people may compare themselves more frequently to each other in terms of the consequences of their actions in the epidemic, eventually creating a deep sense of consequences.

A discussion of information overload cannot be separated from a sharing of the types of information conveyed in information sources, and the impact of information overload on people is certainly different depending on the type of information. In this epidemic, such overload is only possible if there is more information about responsibility and consequences in social media than in other types of information, so it is necessary to understand the main types of information conveyed in the sources that suppress people’s negative behavior and promote awareness of consequences and attribution of responsibility.

Park et al. (3) analyzed information disseminated on social media and found that the most content about “attribution of responsibility” was found in active-epidemic information on Twitters. Studies of the Chinese population have also confirmed that Chinese people often shared information about the epidemic, knowledge and policies on epidemic prevention, expressed their views on responsibility and concerns about the epidemic in social media; and as the epidemic progressed, they also tended to show empathy for patients, blame for those who caused the consequences, and appreciation for protest heroes in social media (4, 62). At the same time, the crisis management culture in Chinese society is state-oriented. In a state-oriented crisis management culture, the people will highly follow the government’s instructions and arrangements to get through the crisis, so when these messages appear in social media, they are conducive to the people’s sense of responsibility and mission, but if they only appear sporadically and do not make enough impression on the people, they must appear repeatedly in large quantities to make users process the information. It is necessary for information to repeatedly appear in large quantities to make users enter into information overload and information cocoon (i.e., only accepting information from information publishers), immersing themselves in these messages and avoiding the interference of other irrelevant information (63). Based on the actual situation, it is reasonable to assume that during the period when China was fighting the epidemic, when information overload occurred, messages on social media may also have repeatedly stimulated people to be aware of the consequences of a worsening epidemic and to never forget the responsibility they need to take. Therefore, the following hypotheses are proposed:

*H5*: Information overload has a positive impact on awareness of consequences.

*H6*: Information overload has a positive impact on the ascription of responsibility.

## 3 Research model and survey

In this study, we formulated relevant hypotheses based on the SOR framework, norm activation theory, and information overload theory, as well as previous research. This study integrates the Stimulus-Organism-Response (S-O-R) framework, Norm Activation Theory (NAT), and Information Overload (IO) theory to construct a systematic explanation of how excessive social media information influences individuals’ anti-COVID-19 behaviors. Specifically, information overload serves as the stimulus (S) in the S-O-R model, triggering cognitive and emotional responses (O) in the form of awareness of consequences and ascription of responsibility (NAT), which in turn activate personal norms that drive prosocial epidemic prevention behaviors (R).”

**Figure 2:**
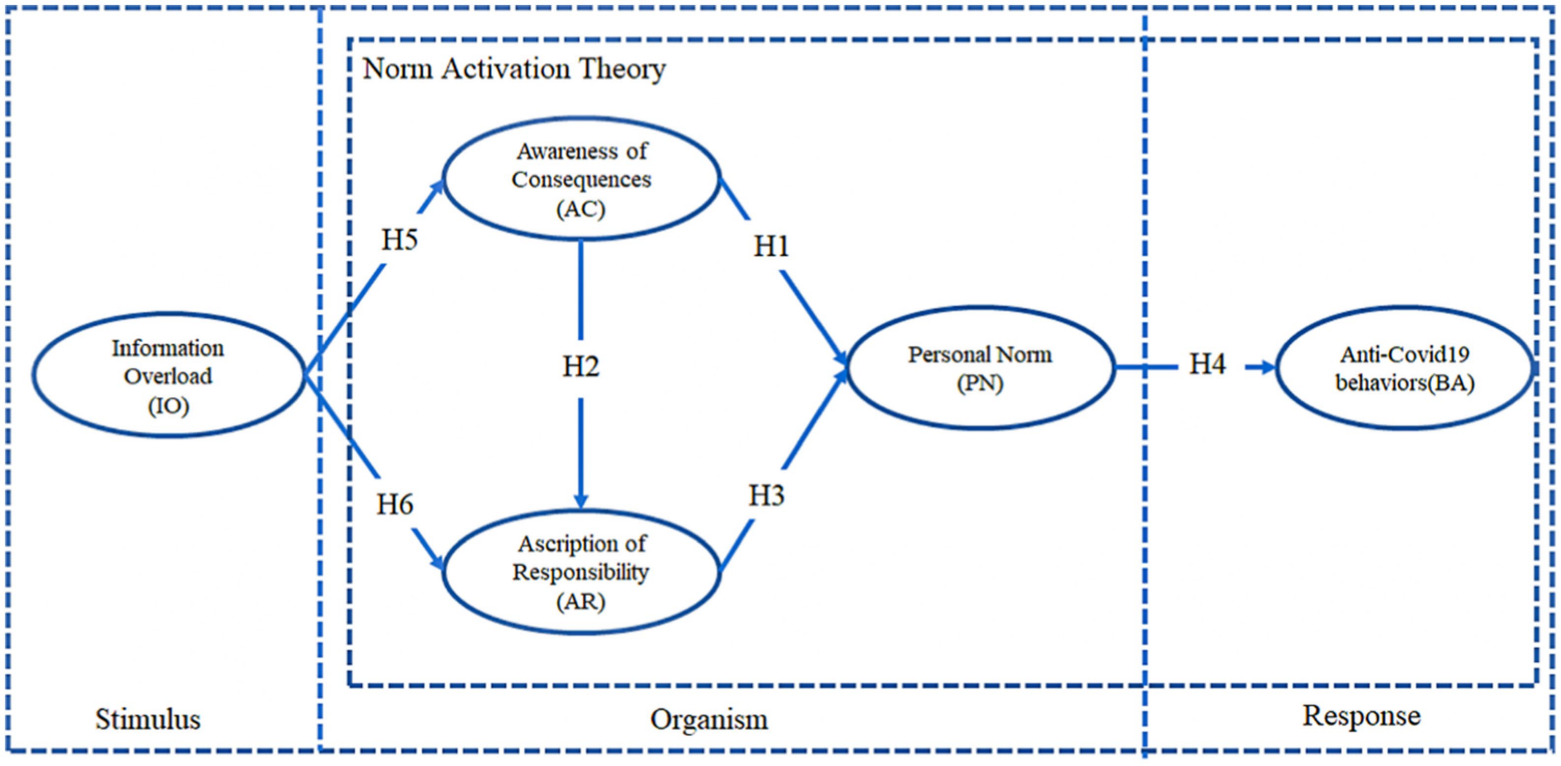
Research mode.

Based on these hypotheses, the research model is shown in Figure 2.

In this study, the questionnaire was designed by modifying the scales of existing studies according to the actual context of the current research. Then, academic experts in MIS research were invited to review parts of the initial questionnaire that were not appropriately formulated and to make improvements to the questionnaire. Please consult Appendix A for the completed questionnaire.

The questionnaire was distributed through SNSs online. All the participants were informed of the following: (1) it was an anonymous questionnaire; (2) the participants clearly knew the content and purpose of the survey and chose to participate freely; (3) the questionnaire did not involve personal privacy information; and (4) the respondents received some minor rewards after completing the questionnaire.

The respondents were recruited in Shijiazhuang, Hebei Province, China, which was classified as a high-risk area for COVID-19 by the Chinese government on January 5, 2021 and received much attention from the Chinese media. From January 5, 2021, the residents of Hebei reached the maximum level of interest for the epidemic in the whole month.

Although the Baidu search index peaked between January 5 and January 9, 2021, information overload (IO) may exhibit a lag effect due to continued social media circulation and algorithm-driven content recommendations (64, 65). Even after search interest declines, users remain exposed to high volumes of related information, leading to prolonged cognitive strain (66). Therefore, the survey in this study was initiated on January 11 to capture the impact of IO beyond the initial search peak. The questionnaire was conducted online from January 11, 2021 to January 30, 2021. All the items in the questionnaire were measured on a Likert 5-point scale. The questionnaire was designed to require a response to each question. Incomplete questionnaires cannot be submitted and if a respondent selected “I seldom use social media,” the questionnaire was automatically eliminated, and no further questionnaires were conducted, thus eliminating omissions and nonrespondents. We distributed 300 questionnaires; after eliminating ineligible and invalid responses, we used 242 valid questionnaires (80.6%) in the study.

The questionnaire also gathered demographic characteristics necessary for the study. There were more male respondents (*n* = 146, 60.3%) than female respondents (*n* = 96, 39.7%). The age distribution was even, with 17.4% of respondents between 18 and 20 years old (*n* = 42), 27.7% between 21 and 30 years old (*n* = 67), 24.8% of respondents between the ages of 31 and 40 (*n* = 60), 14.5% of respondents between the ages of 41 and 50 (*n* = 35), and 15.7% of respondents over the age of 51 (*n* = 38). Most of these individuals had at least a bachelor’s degree, with the largest number reporting their highest educational level of a bachelor’s degree at 52.1% (*n* = 126) and the number of individuals with less than a bachelor’s degree at 63 (26%). Those with master’s degrees totaled 49 (20.2%), and respondents with doctoral degrees accounted for 1.7% (*n* = 4). Most of the respondents had a monthly income between 2000–5000 RMB yuan (approximately USD 285–714), with 171 people accounting for 70.6% of the whole respondents.

In this study, to avoid nonresponse bias, the first and last 20 people who submitted the questionnaire were selected for the paired t test. The results showed that there was no statistically significant difference between the two groups, indicating that nonresponse bias was not a major issue in this study (see Table 2).

**Table 2:** Nonresponse bias test (paired *t* test).

Item	*t*	Sig.
Gender1 –Gender2	1.710	0.104
Age1 – Age2	0.233	0.818
Education1 – Education2	1.073	0.297
Income1 – Income2	1.106	0.283

1, Early submitted; 2, Later submitted.

## 4 Empirical analyses

### 4.1 Analysis methods

There are two types of structural equation models, a covariance-based structural equation model (CB-SEM) and a partial least squares-based structural equation model (PLS-SEM). Our study uses partial least squares structural equation modeling and the corresponding software package smartpls3.0. The PLS-SEM is a multivariate data analysis method for second generation. It is primarily used for exploratory research and allows the independent and dependent variables to be analyzed while ensuring the integrity of all aspects of the relationships between the variables (67).

Partially least squares are useful in social science research for several reasons. First, although PLS-SEM is similar to regression analysis, it also measures the validity and reliability of variables, as well as their relationship to each other and their explanatory power (68). Second, PLS-SEM is useful for prediction since it has a less restrictive sample size than other methods, and it has received much attention from researchers in recent years, especially in the study of management information systems because it allows for the modeling of potential variables without constraints on sample size and relationships between variables (69). Third, PLS-SEM is more suitable for the measurement of complex models than CB-SEM, especially for models with more than six variables (70). Fourthly, PLS-SEM can be used to analyze nonnormally distributed data (70). Finally in contrast to covariance-based techniques (e.g., AMOS), which result in significant loss of indicator variables in pursuit of an acceptable goodness-of-fit (GOF), PLS-SEM has the ability to retain more indicator items to support theory development and measurement. In general, PLS-SEM is more appropriate for theory development than CB-SEM, and most social science research situations can be handled with PLS-SEM instead of CB-SEM (70), PLS-SEM are commonly used in social, economic, and business research.

This study uses a web-based Mardia (71) test calculator^2^ (72) to conduct the multivariate normality analysis for the data. Multivariate normality is one of the criteria for more accurate model prediction (73). Multivariate normality analysis shows that Mardia’s multivariate skewness (*β* =33.864, *p* < 0.001) and multivariate kurtosis (*β* =305.307, *p* < 0.001), which suggests the multivariate non-normality. In summary, this study is an exploratory study, while the data are multivariate non-normality. Therefore, PLS-SEM can handle data of this study very well.

### 4.2 Common method bias

Harman’s single-factor analysis is commonly used in social science research to gauge the likelihood of common method bias (74). A single factor must be extracted according to Podsakoff et al. (75). It means that the survey data are less likely to be affected by common method bias when the variance is less than 40%. The proportion of extracted variables was 33% in this study (less than 40%).

Based on Harman’s single factor analysis, the results are much closer to 40%. Thus, we further performed a bias test using Full-VIF. Kock (76) pointed out that the occurrence of a VIF greater than 3.3 between variables and dump variables in PLS-SEM was proposed as an indication of a model might be contaminated by common method bias. Therefore, if all of the VIFs resulting from full collinearity testing are equal to or below 3.3, the model is free of common method bias. In this study, the full collinearity test among the constructs was lower than 3.3. Therefore, considering the results of the two methods, In this study, method bias was not an serious issue.

### 4.3 Measurement model

Before verifying the structural model, we evaluate the reliability and validity of the model. We evaluated the composite reliability (CR), average variance extracted (AVE), discriminant validity, and outer loading. As shown in Table 3, the composite reliability >0.7 and Cronbach alpha >0.7 indicate that the internal consistency of the data in this study is satisfactory. The AVE > 5 and out loadings>0.7 indicate that the convergent validity of the data in this study is qualified (70). The results of the above analysis indicate that this study has good composite reliability, convergent validity.

**Table 3:** Reliability and validity test result.

Latent variable	Item	Loading	Mean (SD)	Cronbach’s a	CR	AVE
Information overload (IO)	IO1	0.804	3.866 (0.686)	0.751	0.858	0.668
	IO2	0.805				
	IO3	0.842				
Awareness of consequences (AC)	AC1	0.861	3.577 (0.709)	0.828	0.897	0.744
	AC2	0.871				
	AC3	0.855				
Ascription of responsibility (AR)	AR1	0.877	3.539 (0.845)	0.851	0.910	0.770
	AR2	0.876				
	AR3	0.880				
Personal norms (PN)	PN1	0.802	3.545 (0.760)	0.823	0.894	0.738
	PN2	0.894				
	PN3	0.879				
Anti-COVID-19 behaviors (BA)	DC1	0.883	3.477 (0.769)	0.793	0.877	0.704
	DC2	0.841				
	DC3	0.791				

As shown in Tables 4, 5, the discriminant validity of this study was measured using Fornell and Larcker’s Criterion and Heterotrait-Monotrait ratio (HTMT) Test. The Fornell and Larcker’s Test result of each factor was greater than all correlation coefficients (Table 4). The HTMT values (Table 5) between variables were below the 0.85. These results indicate that the discriminant validity of this study scale met the research requirements (70).

**Table 4:** Fornell-Larcker criterion.

	IO	AC	AR	PN	BA
IO	0.817				
AC	0.452	0.862			
AR	0.494	0.270	0.878		
PN	0.291	0.266	0.423	0.859	
BA	0.113	0.245	0.296	0.333	0.839

IO, Information overload; AC, Awareness of consequences; AR, Ascription of responsibility; PN, Personal norms; BA, Anti-COVID-19 behaviors.

**Table 5:** Heterotrait-Monotrait ratio (HTMT).

	IO	AC	AR	PN	BA
IO					
AC	0.572				
AR	0.618	0.323			
PN	0.374	0.318	0.506		
BA	0.151	0.306	0.354	0.398	

IO, Information overload; AC, Awareness of consequences; AR, Ascription of responsibility; PN, Personal norms; BA, Anti-COVID-19 behaviors.

### 4.4 Structural model

A collinearity diagnosis revealed a VIF of less than 5, indicating no problem with collinearity. We tested the overall explanatory power of the structural model and the strength of the path. As shown in Table 6, there is a significant positive relationship between “Awareness of Consequences” and “Personal Norm” (*β* = 0.164, *p* < 0.01), Hypothesis 1 was supported. The relationship between “Awareness of Consequences” and “Ascription of Responsibility” is not significant (*β* = 0.059, n.s.), Hypothesis 2 is not valid. There is a significant positive relationship between “Ascription of Responsibility” and “Personal Norm” (*β* = 0.379, *p* < 0.01), Hypothesis 3 was supported. There was a significant positive correlation between “Personal Norm” and “Anti-COVID-19 behaviors” (*β* = 0.324, *p* < 0.01), Hypothesis 4 was supported. There is a significant positive correlation between information overload and awareness of consequences (*β* = 0.452, *p* < 0.01), Hypothesis 5 was supported. There is a significant positive correlation between “Information overload” and “Ascription of Responsibility” (*β* = 0.468, *p* < 0.01), Hypothesis 6 was supported.

**Table 6:** Hypothesis testing result.

Hypotheses	*β*	STDEV	T-statistics	*p* values	Result
H1:AC -> PN	0.164	0.064	2.553	0.011	Support
H2:AC -> AR	0.059	0.095	0.620	0.535	Reject
H3:AR -> PN	0.379	0.066	5.7564	0.000	Support
H4:PN -> BA	0.324	0.067	4.844	0.000	Support
H5:IO -> AC	0.452	0.058	7.809	0.000	Support
H6:IO -> AR	0.468	0.080	5.857	0.000	Support
Age -> BA	0.017	0.065	0.256	0.798	-
Education-> BA	0.070	0.063	1.098	0.272	-
Income-> BA	−0.026	0.059	0.444	0.657	-
Gender-> BA	−0.040	0.061	0.659	0.510	

IO, Information overload; AC-Awareness of consequences; AR-Ascription of responsibility; PN-Personal norms; BA-Anti-COVID-19 behaviors.

The model fit was checked in this study. Henseler et al. (77) suggest using the standardized root mean square residual (SRMR) as a structural model fit criterion for PLS-SEM. An SRMR value of 0 implies a perfect fit, and an SRMR value of less than 0.08 is generally recommended as an appropriate fit threshold for partial least squares path models (78, 79). Our model has an SRMR value of 0.052, which indicates an appropriate fit of the model.

## 5 Discussion and implication

### 5.1 Summary of results

First, awareness of consequences (AC) and ascription of responsibility (AR) have a positive effect on personal norms, supporting H1 (*β* = 0.164, *p* < 0.01). This result is consistent with norm activation theory (28) and reinforces the idea that when individuals recognize the negative consequences of COVID-19 and attribute responsibility accordingly, they are more likely to internalize personal norms that encourage prosocial, preventive behaviors. In the deterioration of the living environment, awareness of consequences and ascription of responsibility have been identified as important predictors of activating personal norms, such as the purchase of energy-efficient appliances during haze pollution (40). This study reconfirmed the relationship in the context of environmental degradation (COVID-19 pandemic). In the COVID-19 pandemic, there is much information about responsibility on social media (3), and people’s ascription of responsibility and awareness of consequences are more likely to predict the activation of personal norms.

Second, there is no significant positive effect of awareness of consequences (AC) on the ascription of responsibility (AR) (H2 rejected, *β* = 0.059, *p* = 0.535). Such a result is different from existing studies (34, 80). The explanation is that in the prevention and control of an epidemic, the public is more concerned with the consequences of the spread of the epidemic and believes that the spread of the epidemic is a social problem for which they may not be primarily responsible. In addition, previous research on norm activation theory has indicated that awareness of consequences and ascription of responsibility are more likely to play a moderating role in the relationship between personal norms and prosocial behavior (31, 32). Therefore, there is not necessarily a relationship between AC and AR.

Third, personal norms positively influence people’s anti-COVID-19 behaviors (H3 supported, *β* = 0.324, *p* < 0.01). This result is consistent with norm activation theory (26). Numerous empirical studies have also pointed out the strong predictive effect of the activation of personal norms on human prosocial behavior in the context of environmental deterioration (45, 46). This study reconfirmed such results in the context of the COVID-19 pandemic. These results indicate that in during COVID-19, when personal norms are activated, individuals will actively engage in anti-COVID-19 behaviors and contribute to the overall prevention of societal spread of COVID-19.

Fourth, information overload has a significant positive effect on awareness of consequences (AC) (H5 supported, *β* = 0.452, *p* < 0.01) and ascription of responsibility (AR) (H6 supported, *β* = 0.468, *p* < 0.01). A possible explanation is that in an epidemic, people may be in a suboptimal health status, and the effect of information overload is amplified by their unhealthy state, which can easily lead to poor decision-making and dysfunction, causing anxiety or stress (56). When people are limited by their own knowledge, people become overwhelmed by information overload (53). People may enter a state of heuristic information processing where information is processed automatically, independent of individual cognition and ability (24). In the case of information overload, people easily become fatigued, prefer to avoid the information (60), reduce their perception of the cost of response (81) and act unconditionally upon the information perceived. In this way, they believe that they can protect themselves at a minimal cost. Therefore, they are less individualistic and more likely to unconditionally accept social media information and act in compliance with it. In an epidemic, information overload often conveys more information about attribution of responsibility, and deepens the perception of the severity of the situation (3, 81). The information tends to increase people’s perceived risk; for example, in this epidemic, many places have measures to track confirmed patients, and if people are infected, their information will be easily known to society, increasing their perceived privacy risk and perceived moral risk (82). Therefore, when they are overloaded, people may accept and agree with information from social media without thinking which reinforces the perception of the seriousness of the situation. The fear of missing out on information helps people continue to use social media (83), which in turn may exacerbate their information overload, and the cycle continues to deepen their sense of consequences and attributions of responsibility. Moreover, the sense of consequences and attribution of responsibility motivated by information overload is highly correlated with the content and quality of information.

Chinese society’s disaster response culture can be considered a state-dominated disaster response culture, where people strictly follow the requirements of governmental departments in the face of major social disasters. It has been confirmed that in this epidemic in China, social media messages mainly consisted of the transmission of the epidemic situation, the dissemination of epidemic prevention knowledge and policies, the expression of opinions on responsibility and concerns about the epidemic, empathy for patients, blame for those who caused the consequences, and appreciation for protesting heroes (4, 62). So when these messages are repeated to a great degree, they suppress the dissemination of information that is not conducive to active protest and can also put users into information overload. It even stimulates the information cocoon effect, which allows users to immerse themselves in these messages, making it difficult to be influenced by negative information and constantly generating a sense of responsibility and consequence.

### 5.2 Theoretical implications

Several theoretical contributions are presented in this study. First, previous studies have used norm activation theory to validate various prosocial behaviors of people in t specific social problems, while the literature on validation in a wide range of social problems is lacking (34, 39, 40). This study validates the norm activation theory in response to the social prevention behavior of COVID-19, the significant relationship between personal norms and anti-COVID-19 behaviors (H3 supported) confirms that internalized moral norms are powerful motivators in shaping preventive health behaviors, broadening the idea that norm activation theory is also applicable in response to international public health emergencies.

Second, study found that information overload has positive effects. Getting support for H5 and H6 suggests that, within the context of social prevention behaviors in response to international public health emergencies, information overload does not necessarily result in avoidance. Instead, it strengthens the perception of consequences and responsibility, which means that information overload may arguably have a positive effect in leading people toward positive socially beneficial emotions. Such findings are conducive to the enrichment of information overload theory.

Third, this study introduces information overload theory into norm activation theory for the first time, which enriches the antecedents of norm activation theory. The relationship between information overload and prosocial behavior is explained from the perspective of people’s emotions by confirming the influence of information overload on the norm of responsibility and awareness of consequences in norm activation theory. The finding that AC does not significantly predict AR (H2 rejected) also contributes to the literature by suggesting that awareness alone may not be sufficient to activate a sense of responsibility in a pandemic context.

### 5.3 Practical implications

This study also has some practical contributions. First, in response to international public health emergencies, communication regarding awareness of consequences and ascription of responsibility to society must be strengthened. Such communication facilitates the activation of people’s personal norms to benefit society, and such communication also increases the utilitarian value of epidemic prevention information and improves the efficiency of communication.

Second, in responding to international public health emergencies, it is worthwhile to strengthen the push for epidemic prevention information and create some tension in society as appropriate. Thus, society is fully immersed in the atmosphere of epidemic prevention, which has a great effect on reducing individualistic behavior. The international arena offers some successful cases, such as South Korea, where information about the epidemic was pushed to everyone’s cell phone from the national level to the municipal level, regardless of the importance of the information related to the epidemic. Sometimes alerts about the epidemic could reach the public within a few minutes.

Finally, given that AC did not significantly influence AR, policymakers should recognize that simply increasing awareness of COVID-19’s consequences may not be enough to drive responsible action. Future public health initiatives should focus on explicitly linking pandemic awareness to personal responsibility, possibly through targeted interventions, community engagement programs, and behavioral nudges that emphasize individual contributions to collective health outcomes.

### 5.4 Limitations and future research directions

This study also has some limitations. First, because there are many types of information overload, such as communication overload, social overload, information overload, and systemic feature overload, the effects on prosocial behavior must be discussed separately. Second, the gap between the time of this survey and the time of the local COVID-19 outbreak may have affected the validity of this survey due to the development of the epidemic and the weakening of social media attention. Third, the specific content of information delivered in local social networks was not analyzed in this study, and it is recommended that future similar studies use social network analysis (SNA) to target the content of information in social networks (3). In addition, this study was conducted in China, and the sample size was not large enough. More studies are needed in countries with diverse social systems and cultural backgrounds. Finally, this study does not explore the long-term effects of information overload on individuals’ cognitive processing and behavioral adaptation. Future research should investigate whether prolonged exposure to excessive information alters public health information reception, influences long-term adherence to preventive behaviors, or shapes crisis response strategies over time. Longitudinal studies could provide deeper insights into whether cumulative information overload leads to desensitization, selective information processing, or shifts in trust toward public health messaging.

## Data Availability

The raw data supporting the conclusions of this article will be made available by the authors, without undue reservation.

## Footnotes

^1^ http://index.baidu.com

^2^ https://webpower.psychstat.org/models/kurtosis/

## Appendix A

**Table A1:** Measurement scales.

Construct	No.	Item	References
Awareness of consequences (AC)	AC1	Social media helped me understand the impact of COVID-19’s deterioration.	(85, 86)
	AC2	The information displayed on social media has raised my awareness of anti-COVID-19.	
	AC3	The information displayed on social media has reduced my unfavorable behavior to anti-COVID-19.	
Ascription of responsibility (AR)	AR1	Social media tells me that individuals are responsible for the consequences of the spread of COVID-19 as a result of negative behavior.	(86)
	AR2	Social media tells me I have a responsibility to participate in anti-COVID-19 measures.	
	AR3	Social media tells me that everyone must take responsibility for preventing the spread of COVID-19.	
Personal norms (PN)	PN1	I think I am obliged to cooperate with anti-COVID-19 measures.	(85, 86)
	PN2	I had to participate in anti-COVID-19 measures.	
	PN3	I think it is a moral responsibility to take action with anti-COVID-19 measures.	
Information overload (IO)	IO1	I need to spend more time reviewing the information contained in the social media about anti-COVID-19 measures.	(87, 88)
	IO2	There are too many messages about anti-COVID-19 measures in social media.	
	IO3	There is so much information about anti-COVID-19 measures on social media that I cannot consider them all, making the decision more difficult.	
Anti-COVID-19 behaviors (BA)	DC1	I actively cooperate with relevant departments for anti-COVID-19 measures.	(34, 86)
	DC2	I proactively take anti-COVID-19 actions.	
	DC3	Even if it would result in some loss to the individual, I also engage in anti-COVID-19 behaviors.	
